# Supplementary material for: Modeling glioblastoma heterogeneity as a dynamic network of cell states
Source: Mol Syst Biol. 2021 Sep 16;17(9):e10105. doi: 10.15252/msb.202010105 (PMC8444284; doi:10.15252/msb.202010105)
Supplement: Supplementary file 6 — Source Data for Figure 5 [file MSB-17-e10105-s004.zip › Figure5A_sourcedata/GSEA_3071/hallmarks_stateIV.GseaPreranked.1623742551702/HALLMARK_TGF_BETA_SIGNALING.html]

Details for gene set HALLMARK\_TGF\_BETA\_SIGNALING[GSEA]

|  || Dataset | state53071 |
| Phenotype | NoPhenotypeAvailable |
| Upregulated in class | na\_pos |
| GeneSet | HALLMARK\_TGF\_BETA\_SIGNALING |
| Enrichment Score (ES) | 0.2570476 |
| Normalized Enrichment Score (NES) | 1.1041309 |
| Nominal p-value | 0.33483145 |
| FDR q-value | 0.53449565 |
| FWER p-Value | 1.0 |
Table: GSEA Results Summary

  

Fig 1: Enrichment plot: HALLMARK\_TGF\_BETA\_SIGNALING      
 Profile of the Running ES Score & Positions of GeneSet Members on the Rank Ordered List

  

| PROBE | GENE SYMBOL | GENE\_TITLE | RANK IN GENE LIST | RANK METRIC SCORE | RUNNING ES | CORE ENRICHMENT || 1 | ID2 |  |  | 149 | 1.378 | 0.0295 | Yes |
| 2 | WWTR1 |  |  | 277 | 1.081 | 0.0506 | Yes |
| 3 | PMEPA1 |  |  | 373 | 0.939 | 0.0720 | Yes |
| 4 | SPTBN1 |  |  | 387 | 0.918 | 0.1080 | Yes |
| 5 | TGIF1 |  |  | 402 | 0.906 | 0.1433 | Yes |
| 6 | SMAD1 |  |  | 448 | 0.857 | 0.1707 | Yes |
| 7 | APC |  |  | 492 | 0.822 | 0.1970 | Yes |
| 8 | CDKN1C |  |  | 565 | 0.751 | 0.2148 | Yes |
| 9 | BMPR2 |  |  | 677 | 0.673 | 0.2219 | Yes |
| 10 | RAB31 |  |  | 864 | 0.574 | 0.2107 | Yes |
| 11 | HIPK2 |  |  | 896 | 0.563 | 0.2284 | Yes |
| 12 | ARID4B |  |  | 903 | 0.559 | 0.2507 | Yes |
| 13 | BMPR1A |  |  | 1074 | 0.501 | 0.2393 | Yes |
| 14 | SKIL |  |  | 1091 | 0.495 | 0.2570 | Yes |
| 15 | CTNNB1 |  |  | 1728 | 0.344 | 0.1505 | No |
| 16 | NCOR2 |  |  | 2163 | 0.274 | 0.0794 | No |
| 17 | TRIM33 |  |  | 2196 | 0.268 | 0.0846 | No |
| 18 | FNTA |  |  | 2495 | -0.269 | 0.0392 | No |
| 19 | FURIN |  |  | 2938 | -0.324 | -0.0314 | No |
| 20 | TJP1 |  |  | 3113 | -0.348 | -0.0498 | No |
| 21 | FKBP1A |  |  | 3263 | -0.369 | -0.0627 | No |
| 22 | KLF10 |  |  | 3298 | -0.375 | -0.0535 | No |
| 23 | SMAD7 |  |  | 3586 | -0.425 | -0.0902 | No |
| 24 | PPP1CA |  |  | 3654 | -0.436 | -0.0847 | No |
| 25 | PPP1R15A |  |  | 3793 | -0.465 | -0.0914 | No |
| 26 | HDAC1 |  |  | 3827 | -0.471 | -0.0780 | No |
| 27 | ID1 |  |  | 3973 | -0.504 | -0.0844 | No |
| 28 | BCAR3 |  |  | 4626 | -0.750 | -0.1770 | No |
| 29 | ID3 |  |  | 4686 | -0.788 | -0.1552 | No |
| 30 | TGFB1 |  |  | 4781 | -0.846 | -0.1375 | No |
| 31 | SMURF2 |  |  | 4925 | -0.992 | -0.1231 | No |
| 32 | SLC20A1 |  |  | 5094 | -1.277 | -0.1015 | No |
| 33 | SERPINE1 |  |  | 5162 | -1.501 | -0.0513 | No |
| 34 | THBS1 |  |  | 5222 | -1.800 | 0.0129 | No |
Table: GSEA details [plain text format]

  

Fig 2: HALLMARK\_TGF\_BETA\_SIGNALING: Random ES distribution      
 Gene set null distribution of ES for **HALLMARK\_TGF\_BETA\_SIGNALING**
